# Dramatic ENSO related Southwestern Atlantic ecosystem shifts

**DOI:** 10.1038/s41598-025-93080-8

**Published:** 2025-03-06

**Authors:** Simon A. Morley, Fabio Campanella, Emma F. Young, Alastair M. M. Baylis, David K. A. Barnes, James B. Bell, Ashley Bennison, Martin A. Collins, Trevor Glass, Stephanie M. Martin, Paul Whomersley, Andy Schofield

**Affiliations:** 1 British Antarctic Survey, Natural Environment Research Council, Cambridge, UK; 2 Centre for Environment, Fisheries and Aquaculture Science, Lowestoft, UK; 3 Institute for Marine Biological Resources and Biotechnology (CNR-IRBIM), National Research Council, Largo Fiera della Pesca, 2, Ancona, 60125 Italy; 4 South Atlantic Environmental Research Institute, Stanley, Falkland Islands; 5 Tristan da Cunha Government, Edinburgh of the Seven Seas, Tristan da Cunha, UK; 6 Howell Marine Consulting Ltd, Low Hauxley, Morperth, UK; 7 Royal Society for the Protection of Birds, Sandy, UK

**Keywords:** Ecosystem ecology, Physical oceanography

## Abstract

ENSO (El Niño Southern Oscillation) events are becoming more frequent and more intense as climate change continues. Although ENSO effects are known to propagate into the southwest Atlantic, knowledge of how these anomalies result in ecological impacts is yet to be documented. A 3600 km transect in the southwest Atlantic from the Falkland Islands (Malvinas) to Tristan da Cunha during La Niña (March 2018) found a more abundant and speciose seabird assemblage, with more filter-feeding seabird and cetacean species, and a shallower and more concentrated pelagic biomass, than a near identical transect during El Niño (March 2019). Changes in wind and sea surface temperature anomalies correlated, through a one-to-three-month lag, to the peak BEST (Bivariate ENSO Index) index. Stronger winds and greater near surface ocean mixing are for the first time described as mechanisms through which ENSO events can propagate across the southwest Atlantic Ocean basin to alter pelagic ecosystem structure and communities.

Main Text.

**Supplementary Information:**

The online version contains supplementary material available at 10.1038/s41598-025-93080-8.

## Introduction

**Fig. 1:**
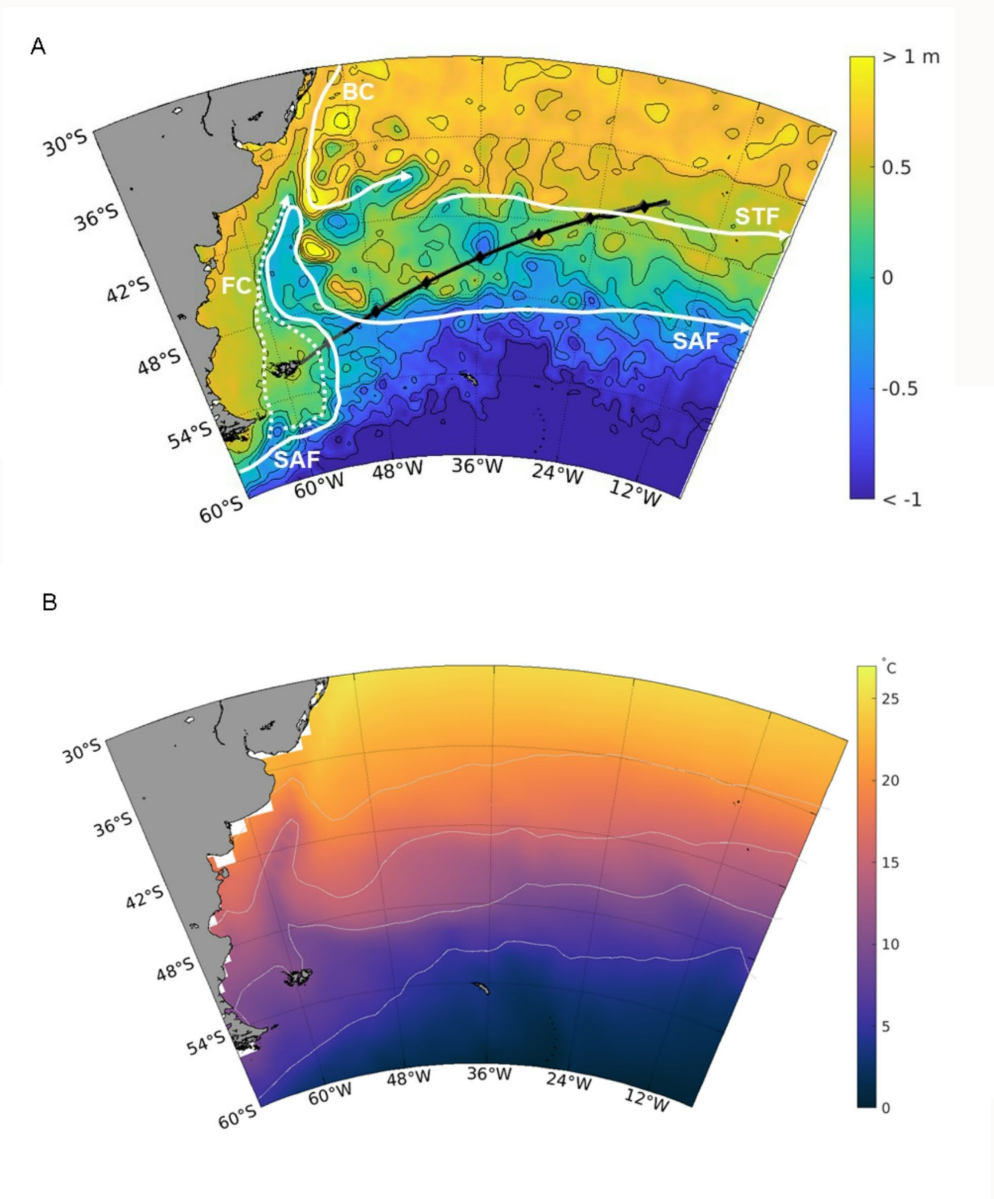
(**A**) Mean absolute dynamic topography for 15 to 21 March 2018 showing the major ocean currents. Cruise tracks, when fisheries acoustics were active (2018 in black and 2019 in grey). The diamonds mark the local midday on each track. A schematic illustration of the mean positions of major fronts and currents in the Southwest Atlantic is shown: Subantarctic Front (SAF), Subtropical Front (STF), Brazil Current (BC), Falkland (Malvinas) Current (FC – dotted white line)^1,2^. (**B**) Mean (1991–2020) March SST for the southwest Atlantic region. Light grey contours are the 5, 10, 15 and 20 °C isotherms.

The southwest Atlantic is a region of complex oceanography, bounded at its southern edge by the Subantarctic Front (SAF) and, to the north, by the eastward flowing South Atlantic Current lying on, or slightly north of, the path of the Subtropical front (STF)^1–4^ (Fig. 1A). The subsequent mixing of cold nutrient rich water with warmer subtropical water creates a region of high productivity, particularly on the Patagonian shelf^5,6^. This contributes to making the region globally important for marine biodiversity, hosting several important seabird and marine mammal feeding and breeding grounds^7,8^.

The southwest Atlantic is a temperate region, with long-term (1991–2020) mean sea-surface temperatures (SSTs) in March that show a broad latitudinal trend of increasing temperatures from south (10 °C) to north (24 °C^1^; Fig. 1B). The complexity of the regional physical and biological systems is thought to make them vulnerable to climate change^9^. Along with the impacts of global warming, periodic climatic events, such as ENSO (El Niño Southern Oscillation) and SAM (Southern Annular Mode) are showing long-term changes. ENSO is a climate pattern involving episodic oscillation of weather patterns in the equatorial Pacific, whilst SAM is the normalized difference in zonal mean surface atmospheric pressure between 40 and 65 °S. With climate change, ENSO is more frequently in an El Niño than La Niña phase^10^, and SAM is more frequently in a higher than lower index state^11^. ENSO events affect global climate and have a strong influence on climate in the southern hemisphere, with effects propagated through global long distance atmospheric and oceanic connections, known as “teleconnections”. A key teleconnection in the south Atlantic is the Antarctic circumpolar wave, which circles the Southern Ocean on an approximately eight year cycle, and has been linked to ENSO^10,12^.

There are multiple temporal signals associated with global teleconnections from east Pacific ENSO events. An immediate atmospherically-forced southwest Atlantic response to ENSO variability has been reported^13^ in addition to a two to three year lag correlated with SST anomalies^14,15^. The Southern Oscillation Index (SOI)^16^ measures the difference in atmospheric pressure between Darwin Australia and Tahiti due to ENSO events. The strength of the SOI is correlated with spring SST and both have been correlated with higher predator breeding success across the south Atlantic and Scotia Sea^12^. Reduced higher predator breeding success is regularly coincident with El Niño throughout the southwest Atlantic^13,14,17^. The strengthening of westerly winds during an El Niño event has been implicated as a driver of both breeding failure of rockhopper penguins (*Eudyptes crestatus*) in the Falkland Islands(Malvinas) and resultant changes to offshore conditions in the Atlantic^18^. This climatic variability can have significant effects on marine ecosystems, some of which are likely to have far-reaching influences into the future.

The most completely described effects of ENSO on ecosystem functioning occur in the southeast Pacific where mass mortalities of benthic species, plankton, fishes and higher predators have been recorded during El Niño events^19–21^. The associated changes in broad scale climatic indices, such as the SOI^21^ and SST anomalies, are often correlated with reduced breeding success of important predators in the southwest Atlantic (e.g., gentoo penguins^13^ and squid^14^). These effects manifest over temporal lags ranging from months to years after a southeast Pacific El Niño event^12,21^, but to date, the links between SST anomalies and coincident ecosystem changes in the southwest Atlantic region are poorly understood, probably due to the complexity of potential interactions^22^.

Two research cruises to support marine management and conservation in the UK overseas territory islands (of St Helena and Tristan da Cunha) departed from Stanley, Falkland Islands (Malvinas, Fig. 1A) in 2018 ^23^ and 2019^24^. These research cruises provided a unique opportunity to complete an almost identical survey transect in consecutive years, during contrasting ENSO phases: a weak La Niña (2018) and a weak El Niño (2019). Using environmental data (SST, Chlorophyll *a*, wind strength and direction) together with species composition (derived from fishery acoustics, seabird and marine mammal observations, and benthic imagery), species assemblages were compared between El Niño and La Niña years to (i) understand the biotic effects associated with such climatic events, and (ii) identify potential drivers of marine ecosystem change in the southwest Atlantic.

## Materials and methods

### Cruise details

Departing on 14 March 2018 aboard the RRS James Clark Ross^23^ and 12 March 2019 aboard RRS Discovery^24^, two UK Government funded cruises transited from the Falkland Islands (Malvinas) to Tristan da Cunha two calendar days apart in consecutive years (Fig. 1A).

NOAA’s Oceanic Niño Index (ONI)−which measures the rolling three-month temperature anomalies in the surface waters of the east-central tropical Pacific,−was used to characterize El Niño and La Niña phases. This index indicated that March 2018 was seven months into a La Niña (ONI, -0.5 to -1.0, Sep 2017 to May 2018), while March 2019 was seven months into an El Niño (ONI, + 0.7 to + 0.8, Sep 2018 to July 2019).

### Satellite-derived oceanography and wind data

We used a number of open access datasets to characterize the marine environment of the southwest Atlantic. For comparisons with cruise data, high-resolution environmental data capable of resolving mesoscale spatial features were obtained. All analyses and data visualization were performed in Matlab R2022b and used the M_Map mapping package^25^, together with the cmocean^26^ color maps for visualization.

DUACS delayed-time altimeter gridded maps of sea surface heights (SSH) and corresponding geotrophic velocities over the global ocean at 0.25° horizontal grid resolution were obtained from the Copernicus Marine Data Store (10.48670/moi-00145). Sea surface temperature (SST) data were similarly obtained from the Copernicus Marine Data Store; the OSTIA^27^ global SST reprocessed product provides daily gap-free maps of foundation SST (free of diurnal variability) at 0.05° horizontal grid resolution, using in-situ and satellite data.

Monthly SST anomaly data^28^ on a 1° horizontal grid, relative to a 1991–2020 climatology, were obtained for January 1982 to December 2022 from the IRI/LDEO Climate Data Library (http://iridl.ldeo.columbia.edu/SOURCES/.NOAA/.NCEP/.EMC/.CMB/.GLOBAL/.Reyn_SmithOIv2/.monthly/.dataset_documentation.html). These data were used for analysis of long-term variability in regional SST anomalies and to visualize SST anomalies for the months preceding and contemporaneous with the cruises.

Chl *a* data were obtained as monthly composite data at 4 km horizontal grid resolution from MODIS (http://oceandata.sci.gsfc.nasa.gov). The algorithm returns the near-surface concentration of Chl *a* in mg m^− 3^, calculated using empirical relationships derived from in situ measurements of Chl *a* and remote sensing reflectances^29^. Monthly data were chosen to minimize the impact of cloud cover obstructing the images.

Monthly mean 10 m winds were downloaded from the ECMWF Reanalysis v5 (ERA5) atmospheric reanalysis, provided by the Copernicus Climate Service^30^. Mixed layer depths (MLDs) were obtained for March 2018 and 2019 for the southwest Atlantic region from a database combining Argo profiles and a hybrid algorithm for detecting the MLD (https://mixedlayer.ucsd.edu/)^31^. The irregularly distributed data were gridded onto a regular 0.5º horizontal grid using natural interpolation.

Differences in large-scale SST and Chl *a* were visualised by plotting the differences between the observed fields (2019 minus 2018); for calculation of SST differences, the data were temporally averaged over 12–21 March of each year, a period chosen to encompass the cruise periods. For comparison with seabird and cetacean sightings, satellite-derived SST, Chl *a*, and MLD were extracted for the relevant locations and times.

### Underway acoustics

Multifrequency acoustic data were collected using a hull-mounted Simrad EK60 echosounder (18, 38, 70, 120, 200, and 333 kHz). The echosounders were calibrated using a 38.1 mm tungsten carbide sphere following the standard sphere method^32^. The calibration performed on the RRS *Discovery* highlighted a malfunctioning of the 18 and 38 kHz transducers, precluding the use of those frequencies. For this reason, the main frequency used for the analysis was 70 kHz (70 kHz was also used for the 2018 data for consistency). The settings and calibration parameters of the acoustic equipment used in the two expeditions are listed in Supplementary Tables S2 and S3. The acoustic data from the surface to a maximum depth of 600 m (below which the 70 kHz data were affected by absorption) were cleaned and processed using the software Echoview v10. The area immediately below the transducers (10 m below) was excluded from the analysis because it was affected by the near-field effect and surface noise (e.g., surface bubbles). Background noise, pulse noise and attenuated signals were removed using a series of tools integrated in the Echoview software. Nautical Area Backscattering Coefficient (NASC), was exported from the “clean” echograms and used for further analyses. NASC, also referred to as backscatter, was considered a proxy for biomass. The horizontal sampling unit used to export the NASC was 1 nm (nautical mile).

In order to discriminate different classes of acoustic target, a combination of thresholding and the difference in mean volume backscattering strength (DB differencing) was also used^33^. The analysis consisted of two steps: (i) Thresholding - Mean Volume Backscattering Strength (MVBS) at 70 and 120 kHz was summed, and the resulting echogram was thresholded in order to separate two broad classes of targets (fish vs. plankton; Table S4). The difference in variability between fish and zooplankton was used to enhance the contrast between both types of organisms. The use of this approach is helpful when there is a high density of gas-bearing plankton that can easily be mistaken for fish if only dB-differencing is used. The threshold value used for the data collected during the day was empirically chosen at -140 dB. Values above the threshold (> -140dB) were classified as fish and values below the threshold were classified as plankton. A Boolean mask was then created to assign the backscatter to fish and plankton; (ii) DB differencing – the fish and plankton categories were further separated into four additional classes (fish with swimbladder, fish without swimbladder, fluid-like plankton, gas-bearing plankton). Identification of these classes was based on the differences of MVBS measured at 120 and 70 kHz (ΔMVBS120–70, Supplementary table S3). The NASC for the “fish” and “gas-bearing” plankton categories was exported at 70 kHz using a minimum Sv (Volume backscattering strength, dB re 1 m^− 1^) threshold value of − 70 dB. The NASC for the “fish without swimbladder” and “fluid-like plankton” categories was exported at 120 kHz using a minimum Sv (Volume backscattering strength, dB re 1 m^− 1^) threshold value of − 85 dB.

The spatial patterns of distribution of the different scatter categories were investigated using a set of spatial metrics namely estimated center of gravity, equivalent area and inertia calculated in a cell of 1 nm (horizontally) by 200 m (vertically). The center of mass is the mean location of the backscatter in the water column; the inertia is a measure of the dispersion of the backscatter around its center of gravity; the equivalent area is an individual-based index of the area occupied by the backscatter and it is a measure of the evenness^34,35^. The spatial metrics were estimated using the daytime data only to exclude the effects of vertical migrations within the water column. The spatial metrics and the NASC were grouped in 5 º longitudinal classes and the differences between the two years were investigated graphically and statistically using the non-parametric Mann-Whitney U test.

### Seabird observations

During daylight hours, seabirds were counted by a single experienced observer within 300 m from bow to beam on one side of the vessel following European Seabirds At Sea (ESAS) recommended protocols^36^. Following ESAS recommendations, for the South Atlantic away from coastal influence, only seabirds in flight were recorded. Observations, for both seabirds and marine mammals, were continuous throughout daylight hours (over 900 km of observations on each cruise; Table S5) and the total number of seabirds of each species were counted during every 10-minute period as the vessels steamed at an average speed of 10 knots. Seabirds circling the stern and therefore following the ship were eliminated by a check to the stern during each period. Counts were entered directly into the BirdLasser app which records a GPS co-ordinate for every record (https://www.birdlasser.com/).

### Mammal observations

During daylight hours observations were made by two experienced marine mammal observers, in an 180° arc, either side of the vessel, using handheld binoculars. Standard methods use two marine mammal observers, with each covering a 90º arc on one side of the vessel. Observers identified species and recorded low, high and best estimates of group sizes, while the vessel continued along its course. The best estimate was reported as the group size. Since these were not dedicated marine mammal surveys, no deviations from the transit track were made, therefore, depending on sea state, individuals were visible for approximately 20 min. Sampling effort is detailed in Table S5.

### Statistical analysis

Correlations between environmental variables (magnitude of wind components, wind speed and SST anomaly) and the Bivariate El Nino-Southern Oscillation Index (BEST) involved calculation of Pearson correlation coefficients, with statistical significance inferred for *p* < 0.005. Analyses encompassed the full period of temporal overlap in the data, specifically January 1982 to December 2022. Correlations with temporal lags at one-month intervals, spanning up to four years, were conducted with the BEST index preceding the environmental variables, to find the temporal signature of correlations.

To test for differences in seabird assemblage the data were firstly visualized as shade plots in Primer v.7 ^37^. These showed that fourth root transformation was most appropriate to reduce the dominance of a few very abundant species, before non-metric multi-dimensional scaling analysis (nMDS). The main species driving the differences between the two years in the whale and seabird assemblages were detected using an analysis of similarity percentages (SIMPER). The combination of the four factors, wind strength, MLD, SST, Chl *a* and NASC, that were most strongly correlated with the seabird and marine mammal assemblages were calculated using the BEST Biota-environment (BIOENV) procedure (Primer v.7).

## Results

### Sea surface oceanography and wind data

**Fig. 2:**
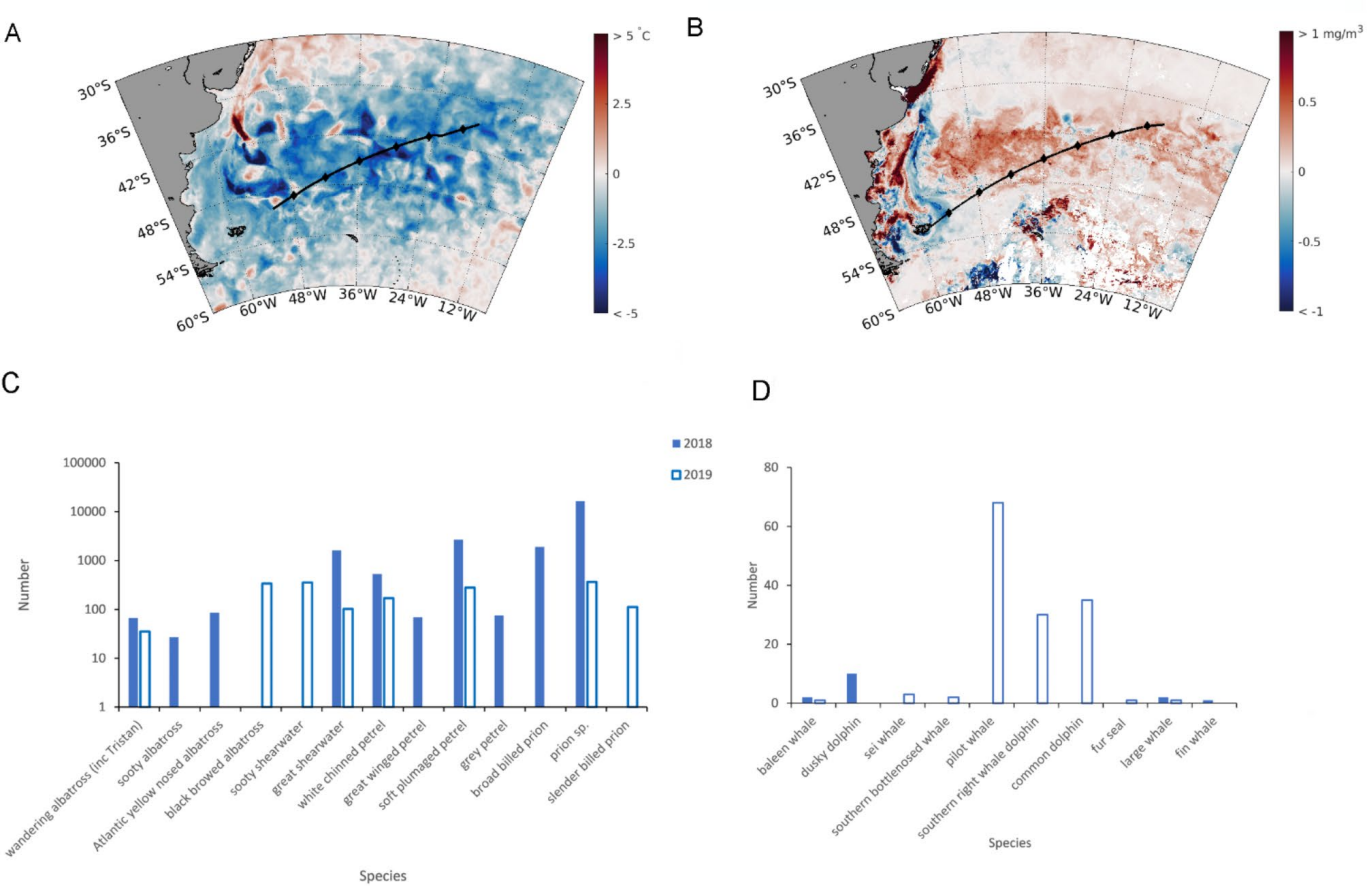
Differences in (**A**) satellite derived SST (2019 relative to 2018) and (**B**) satellite derived chlorophyll *a* (2019 relative to 2018), (**C**) bird and (**D**) marine mammal observations during the two cruises in March of 2018 and 2019. The cruise tracks, when fisheries acoustics were active, are shown in (**A**) 2018 and (**B**) 2019, with local midday labelled with diamonds. The total numbers of the most abundant 10 species of birds (C) and all observed species of marine mammals (D) are plotted (2018 = solid bars, 2019 = open bars).

SSTs in the southwest Atlantic region differed between early 2018 and 2019 (Fig. S1), as 2019 was slightly colder than 2018 across the majority of the cruise track and most of the region (Fig. 2A). Mean SSTs in the study region were 14.4 °C in 2019 (El Niño) and 15.3 °C in 2018 (La Niña). There was a clear seasonal progression of the mean SST anomalies from December to March, when the strongest positive (2018) and negative (2019) anomalies were detected (Fig. S1). There was a significant negative correlation between SST anomaly and the BEST index, peaking at a time lag of three months (BEST preceding SST anomaly; Fig. S2 and S3) and with the strongest correlation in the east of the region (*r* = -0.45, *p* < 0.001). There were significant positive correlations between BEST and regional winds for time lags of one to two months (BEST preceding wind variable). The strongest correlations were in the northeast for wind speed (*r* = 0.18. *p* < 0.001) and the westerly wind component (*r* = 0.20, *p* < 0.001), and in the southwest of the region for the southerly wind component (*r* = 0.18, *p* < 0.001; Fig S4). Over the wider southwest Atlantic region, deeper mean MLDs were observed in 2019 (50.5 m), than in 2018 (38.6 m).

The colder year (2019) was associated with increased Chl *a* standing stock across most of the cruise track, except for the region between the Falkland Islands (Malvinas; 59 °W) and approximately 54 °W, where the Chl *a* was markedly reduced (Fig. 2B). The monthly progression of summer Chl *a* showed a strong peak between 48 and 42 °S in January 2018 (Fig. S5), weakening through February and March. By contrast, productivity south of 48 °S was higher in December 2018 and January 2019 relative to the previous year, with the band of higher productivity developing further north (48 to 42 °S) in February and March 2019, corresponding to the time and location of the cruise track.

### Differences between assemblages

**Fig. 3:**
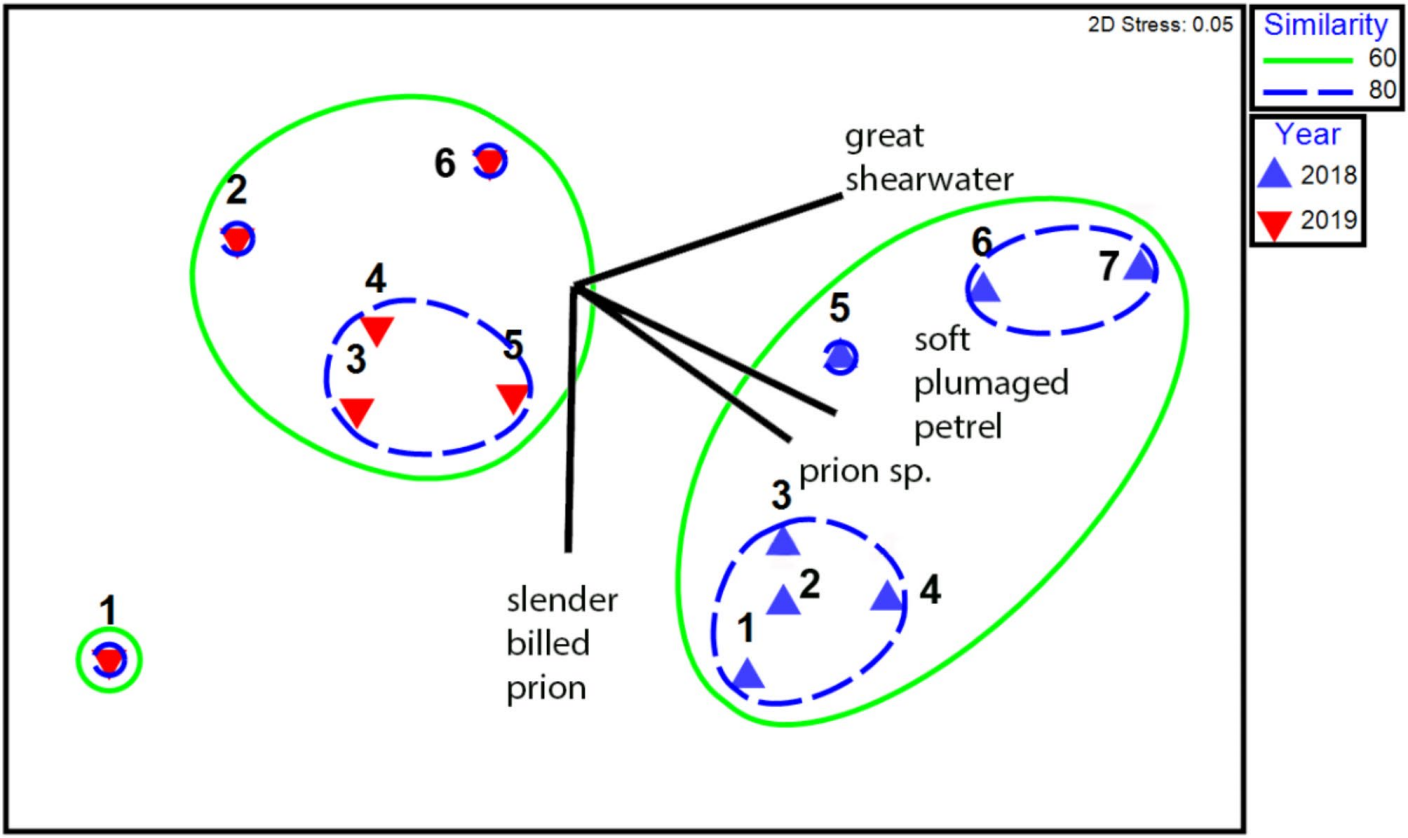
Non-metric multi-dimensional (nMDS) scaling of bird and marine mammal species abundance during the passage from the Falkland Islands (Malvinas) to Tristan da Cunha during March 2018 (RRS *James Clark Ross*) and 2019 (RRS *Discovery*). The west to east progression of sites 1–7 in 2018 and 1–6 in 2019, match the observation days reported in Fig. S6. Ellipses indicate Bray-Curtis similarities of 60 and 80%. The vectors indicate the four species constituting the greatest difference, and the direction of this difference, between the cruises, contributing 32.5% of the dissimilarity from a SIMPER analysis. nMDS was calculated in Primer v.7 from a Bray-Curtis similarity matrix after shade plots confirmed that a fourth root transformation was required to reduce the influence of abundant species (Primer v.7).

There were clear differences between the composition of seabird and whale assemblages recorded in 2018 and 2019 (Figs. 2C, D and 3; Fig. S6; Table S1) and from south to north along the research cruise track (Fig. 3). In 2018, the numbers of birds were an order of magnitude higher (Total *n* = 31,015) than in 2019 (Total *n* = 1,975), and the species composition also differed. In 2018, flocks of surface-feeding, smaller seabird species dominated, such as the prions (*Pachyptila* sp., *n* = 16390; slender billed prion, *Pachyptila belcheri*, *n* = 3304; broad billed prion, *Pachyptila vittata*, *n* = 1904 and Antarctic prion, *Pachyptila desolata*, *n* = 684), soft plumaged petrels (*Pterodrama mollis*, *n* = 2662) and great shearwaters (*Ardenna gravis*, *n* = 1614). Simper analysis showed that soft plumaged petrel (10.3%) and prion sp. (8.9%) were the main species differences (dissimilarity) between 2018 and 2019 (Fig. 3). Great shearwaters were more common in the northern seabird assemblage and slender billed prions in the southern bird assemblage, responsible for biggest difference between assemblages, 4.9% and 6.0% respectively (Fig. 3, Fig. S6). In 2019, there were overall fewer seabirds recorded with a greater proportion (although lower numbers) of larger seabird species, that take larger prey, notably wandering (*Diomedea exulans*, *n* = 35), sooty (*Phoebetria fusca*, *n* = 7) and black browed (*Thalassarche melanophris*, *n* = 338) albatross. This interannual difference in the seabird assemblage was also mirrored in the marine mammal observations, showing a higher proportion of filter feeder baleen whales in 2018 (4 out of 5 sightings: 33%, 5 out of 15 individuals), while almost exclusively toothed whales in 2019 (6 out of 9 sightings: 96%, 136 out of 141 individuals) (Fig. 2D).

Differences in the seabird and marine mammal assemblages between years (BIOENV, Rho = 0.28, *p* = 0.01; Table S1) were most strongly correlated with the combination of four factors: wind strength, Chl *a*, MLD, and SST (R^2^ = 0.58). The next strongest correlation was with the combination of two factors: wind strength and Chl *a* (R^2^ = 0.49) and the third strongest correlation was with the single factor, wind strength (R^2^ = 0.48).

**Fig. 4:**
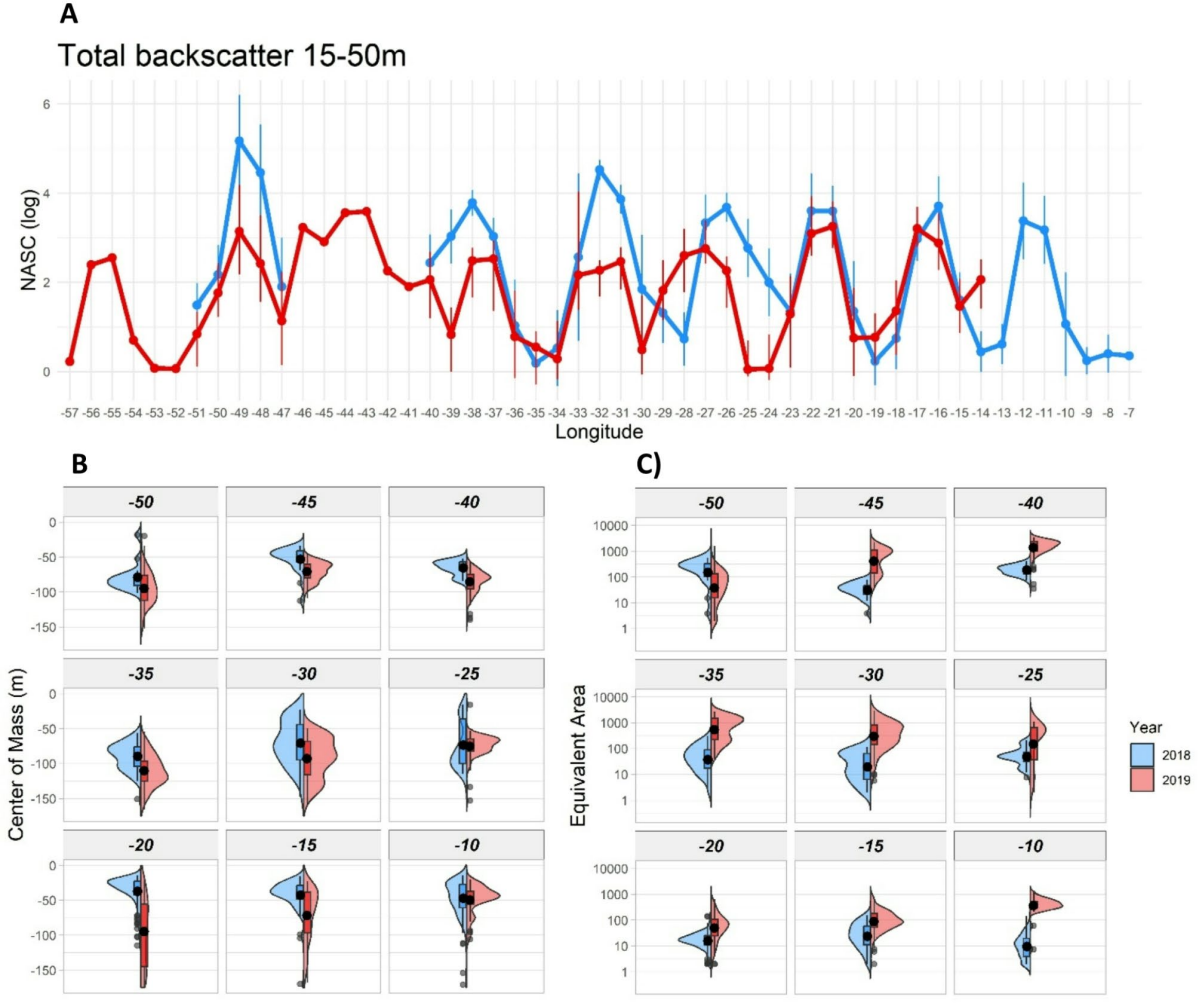
Total NASC (Nautical Area Backscattering Co-efficient) at 70 kHz, recorded during daytime, as a proxy for pelagic biomass during the transit from the Falkland Islands (Malvinas) to Tristan da Cunha on both the JCR (2018) and DY100 (2019) cruises. (**A**) Total NASC between 15 and 50 m, (**B**) Depth distribution of centre of mass of total NASC per 5º of latitude and (**C**) equivalent area per 5º of latitude (a log-transformed index of the evenness of NASC).

Total estimated pelagic biomass (NASC) was on average 2.4 times greater in the top 50 m in 2018 than 2019 (NASC, 15 to 50 m; Fig. 4A). Total pelagic biomass was significantly higher at 25, 35, 40, 45 and 50 m in 2018 than 2019 (Table 1). This represented a redistribution of biomass into shallow water, as over depths of 15 to 200 m the total backscatter was very similar between years (Fig. S7, Table 1). The analysis of the center of mass also confirmed this trend indicating a significantly shallower center of mass of the total backscatter in 2019 compared to 2018 along the ship track (Fig. 4B). The higher density of backscatter in 2018 in the 15 to 50 m depth layer was mainly driven by the gas-filled plankton and fish with swimbladders (Fig. S8). In general, the aggregative behaviour of the pelagic biomass was also significantly different, more concentrated and localized in 2018, while more disperse in 2019. Equivalent area was lower in 2018 than 2019 for most of the ship track indicating the presence of more concentrated and localized aggregations compared to a more disperse pattern in 2019 (Fig. 4C). The analysis of the inertia showed some differences between the two years even though differences were less evident than the rest of the spatial metrics (Fig. S9). In particular, the inertia in 2018 was lower than 2019 for most of the ship track indicating a lower variability in the vertical distribution of the total backscatter. During the 2018 transit, stripes of krill were observed along the western portion of the transect collecting in windrows at the surface, supporting the higher concentration of pelagic biomass detected closer to the surface.

**Table 1:** Results of the Mann-Whitney U test comparing the total NASC and the Spatial metrics between the 2018 and 2019 surveys. Values in bold indicate significant differences between the two years.

	*p*-value (Mann-Whitney U Test)			
Longitude	NASC	Inertia	Equivalent Area	Center of mass
-10	0.160	**< 0.001**	**< 0.001**	0.740
-15	0.274	**< 0.001**	**< 0.001**	**< 0.001**
-20	0.950	0.035	**< 0.001**	**< 0.001**
-25	**< 0.001**	0.077	**< 0.001**	**< 0.001**
-30	0.974	**< 0.001**	**< 0.001**	**< 0.001**
-35	**< 0.001**	**0.005**	**< 0.001**	**< 0.001**
-40	**< 0.001**	**0.003**	**< 0.001**	**< 0.001**
-45	**< 0.001**	**< 0.001**	**< 0.001**	**< 0.001**
-50	**< 0.001**	0.758	0.769	0.210

## Discussion

This study identified strong differences in the effects of El Niño and La Niña events on marine assemblages, which was manifested through regional winds, Chl *a* as a proxy for productivity, MLD, and SST across a broad region of the southwest Atlantic. These were correlated with changes in surface food web structure, through quantified differences in seabird and marine mammal assemblages, and near surface NASC. Although this was only a comparison between two years, it provides the first potential mechanistic pathway between ENSO fluctuations and changes in surface food web structure at an ocean basin scale. This suggests similar mechanisms to the southeast Pacific^38^ could be associated with ENSO effects in the southwest Atlantic, and that these effects can propagate over relatively short, one to three month timescales.

The research cruise tracks in this study were completed seven months into weak, but consecutive, La Niña and El Niño events. In 2019, winds over the southwest Atlantic had stronger westerly and weaker southerly components, and were stronger, compared to 2018 (spatially-averaged March mean wind speed 4.78 vs. 3.63 m s^− 1^). Concurrently, deeper mean MLDs were observed in 2019, compared to 2018^31^, suggesting that the center of mass of the total backscatter was deeper and upper water column and surface biomass was spread over a greater depth in 2019. This study highlights how ENSO fluctuations could propagate through to structural changes in upper ocean food webs, providing the first indication of atmospheric and oceanic variability associated with El Niño generating ecological impacts on higher trophic levels in the southwestern Atlantic. The current study found ENSO correlations with changes in primary production, which was previously found to be a more important correlate of regional assemblage structure on seamounts than temperature^39^. However, many of the individuals that dominate continental shelf benthic biomass are sessile and old, so the detection of any productivity-associated change on the benthos may take decades^40^. Thus, it is unsurprising that we found rapid ecological responses at the surface whilst none were apparent on the Tristan da Cunha seamounts during this same period (re-analyzed from^41^).

Interpretation of the difference in seabird and marine mammals can require more caution. While birds were observed feeding at the surface and shallow sub-surface, it is not always possible to infer from visual observations if animals are feeding or travelling. There were clearly higher numbers of seabirds in 2018 and more sightings of Odontocetes in 2019, which are valuable records for this infrequently surveyed region and could be related to differences in pelagic feeding conditions. Seabird distributions are known to correlate with oceanic properties^42^ and mesoscale features^43,44^. Seabirds are known to adopt short-term changes in distribution in relation to climatic events^45^, and so it is likely that ENSO-related changes in species assemblage, abundance, and distribution here are responses to the altered environment, likely related to prey availability^46^. The first of the two years (2018) documented a community assemblage of smaller prions and petrels feeding from the surface or with shallow dives (< 10 m), on a variety of prey including zooplankton, squid, and fish^47,48^. This is supported by the higher amount of backscatter in the surface waters in 2018, suggesting higher densities of prey near the surface. Such patches of pelagic biomass with higher density might have also increased the foraging efficiency of the predator assemblages in 2018 as these species rely on the detection of such prey patches to feed optimally^49–51^.

The interannual differences in seabird and cetacean species assemblage consisted of a shift to predominantly larger albatross, and toothed whales, in 2019, which feed on larger prey items notably including squid^52,53^. The ENSO variability documented between the two years was correlated with considerably different species assemblages and abundance, which implies large interannual shifts in the distribution of these species. Even though in this work we did not directly evaluate the pelagic species community, we acoustically observed an increase of the gas-filled plankton acoustic category, which can include for example siphonophores and other gelatinous organisms, observed in the sub-surface layer in 2018. These species feed predominantly on plankton, indicating a similar pattern to the planktivorous seabird species^54^.

Seabird reproductive success has been linked to El Niño events across the Southern Hemisphere. In western Australia, lesser and brown noddies, sooty terns, and wedge-tailed shearwaters all showed reduced breeding success during ENSO events, which was associated with locally reduced availability of key prey species^55^. Furthermore, large sub-tropical dipole anomalies in the South Atlantic and Indian Ocean have been linked to reduced breeding success in Southern Ocean king penguin populations, largely due to shifts in their feeding grounds, increasing foraging distances from breeding colonies^56^. The two years of survey documented here were at the end of the Austral summer, seven months into either La Niña or El Niño events and therefore likely represent the prevailing summer conditions and distribution of breeding seabirds. If these shifts in distribution led to longer foraging trips during the breeding season, then this study provides a sequence of linked effects of ENSO that could impact their breeding success in the southwest Atlantic^13,14^.

The El Niño effects on oceanic food webs we describe for the southwest Atlantic are similar processes to those documented for the southeast Pacific, particularly those on the near-shore ecosystem along the coast of Peru^38^. In the eastern Pacific, a slowing of the easterly trade winds is associated with a reduction in the associated upwelling, and results in warmer SSTs^57^. The lack of upwelling reduces the flow of nutrients into the upper ocean, in turn reducing primary productivity and thus the supply of particulate organic carbon to the sea floor and communities living there^20,56,58^. These changes correlate with reduced primary production and die-offs of shallow water benthic algae and phytoplankton, which are thought to propagate through to mortalities of many of the fish and seabird predators that rely on the base of this ecosystem^56^. While populations on this Pacific coast are adapted to these episodic climatic events, the predicted increase in strength and frequency of El Niño^11,19^ is likely to combine with other anthropogenic stressors (exploitation, habitat and climate change) to increase the pressure on already vulnerable populations.

The time series of satellite-derived SST anomalies, together with the correlations between BEST, SST anomalies and winds indicate that even though measurements were only recorded during two five-day research cruises, they were representative of regional ENSO-derived anomalies^59^. The time series from December until the research cruise in March showed the development of the SST anomalies, with the maximum difference between years recorded during March. The southwest Atlantic pattern associated with Chl *a* is less clear but as a proxy for surface productivity, surface standing stock of Chl *a* generally reduced from December to March in 2017/2018, in line with seasonal expectations. Long-term variability in primary productivity does emerge as a key driver of regional seamount biological richness^31^ but we would not expect to (and did not) detect this across just two years of benthic imagery^40^. Although Chl *a* was also generally reduced in 2018/2019, a band of Chl *a* productivity remained along the cruise track in March of the El Niño year, which may be related to the stronger winds, deeper MLD, and thus enhanced nutrient availability in 2019. Measurements from satellites only measure the near surface Chl *a* (shallowest 10 m), but the NASC data give an indication of what is happening, more generally, in the water column and reinforces that there were differences in how much food was available, for secondary and higher consumers within the surface layers in 2018 than 2019. This difference correlated with the abundance of filter-feeding higher predators, and as discussed above, likely indicates they have changed foraging location.

The predicted consequences of climate change include both a warming climate and increased extremes of climate variability. In the Pacific, climate change is altering the distribution of ENSO-related SST anomalies and El Niño events are expected to increase in intensity as central Pacific SST gradients increase^60,61^. This intensity is expected to be transmitted through connected oceanographic and weather patterns to the South Atlantic, increasing ecosystem variability and potentially increasing the frequency of poor breeding years of seabird species that are already classified as vulnerable. This study indicates that SST anomalies can be an indicator of wider changes in southwest Atlantic pelagic ecosystems driven by ENSO variability and has, for the first time, described a mechanistic link that can explain how ENSO-driven changes in wind direction and strength, with subsequent impacts on mixed layer depths, can propagate through to ecosystem level changes that have the potential to impact already vulnerable populations of higher predators. Clearly, further studies, capturing a wider range of ENSO conditions are required to further investigate the consistency of this response. The evident short-term changes we observed during the ENSO events provide us with an opportunity to better understand these ecological processes and potentially predict ecosystem changes under longer-term climate change, either by an increased frequency of ENSO events or from the broader impacts of global warming. This knowledge could help inform conservation strategies and policy decisions aimed at mitigating the effects of climate change on the southwestern Atlantic ecosystem.

## Electronic supplementary material

Below is the link to the electronic supplementary material.

Supplementary Material 1

## Data Availability

All data are available at: Morley, S. A., Campanella, F., Baylis, A. M. M., Barnes, D. K. A., Bell, J. B., Bennison, A., Collins, M. A., Glass, T., Martin, S. M., Whomersley, P., Young, E. F., & Schofield, A. (2024). Fisheries acoustic data, whale and bird data from two transits from the Falkland Islands to Tristan da Cunha during March 2018 (RRS James Clark Ross) and March 2019 (RRS Discovery) (Version 1.0) [Data set]. NERC EDS UK Polar Data Centre. https://doi.org/10.5285/DB06C590-4F6D-4A8A-9B8C-CE45204103C1.
