# Supplementary material for: Dramatic ENSO related Southwestern Atlantic ecosystem shifts
Source: Sci Rep. 2025 Mar 6;15:7917. doi: 10.1038/s41598-025-93080-8 (PMC11885653; doi:10.1038/s41598-025-93080-8)
Supplement: Supplementary file 1 — Supplementary Material 1 [file 41598_2025_93080_MOESM1_ESM.docx]

Supplementary information for:

Dramatic ENSO related southwestern Atlantic ecosystem shifts

Simon A. Morley^1^* Fabio Campanella^2,3^, Emma F. Young^1^, Alastair MM Baylis^4^, Dave KA Barnes^1^, James B. Bell^2^, Ashley Bennison^1^, Martin A Collins^1^, Trevor Glass^5^, Stephanie M. Martin^1^, Paul Whomersley^6,2^, Andy Schofield^7^.

^1^ British Antarctic Survey, Natural Environment Research Survey, Cambridge, UK.

^2^ Centre for Environment, Fisheries and Aquaculture Science, Lowestoft, UK.

^3^ Current address: National Research Council,Institute for Marine Biological Resources and Biotechnology (CNR-IRBIM), Largo Fiera della Pesca, 2, 60125 Ancona, Italy

^4^South Atlantic Environmental Research Institute, Stanley, Falkland Islands(Malvinas).

^5^ Tristan da Cunha Government, Edinburgh of the Seven Seas, Tristan da Cunha

^6^ Howell Marine Consulting Ltd., Low Hauxley, Morperth, UK

^7^ Royal Society for the Protection of Birds, Sandy, UK.

*Corresponding author: Simon A Morley.

Email: smor@bas.ac.uk

This file contains:

8 figures

4 tables

**
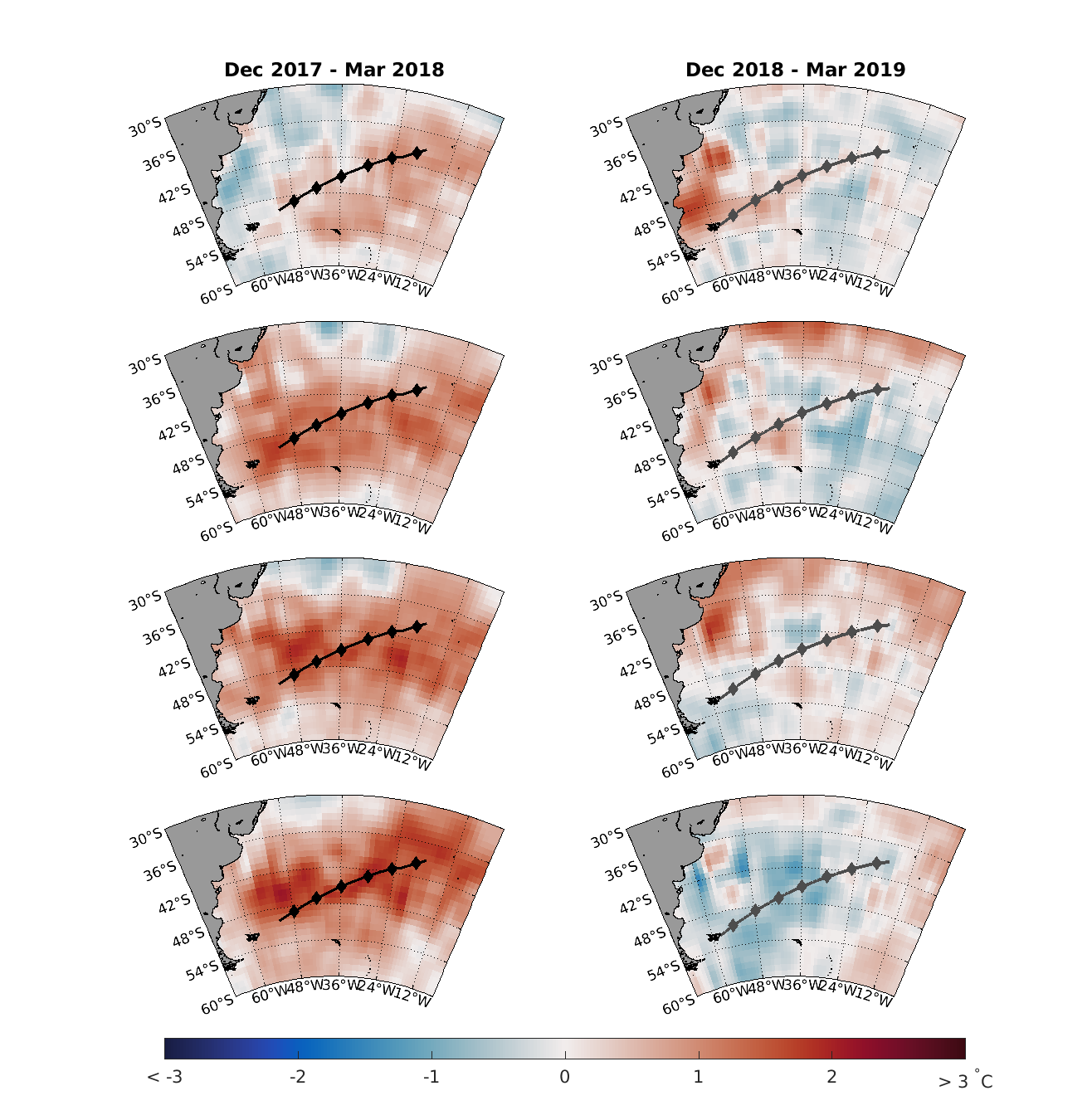
**

**Figure S1.** Monthly progression of mean sea surface temperature anomalies (relative to 1991-2020 climatology) prior to and contemporaneous with the cruises in 2018 and 2019 (see methods for details). Cruise tracks, when fisheries acoustics were active, are shown as black (2018) and grey (2019) lines, with diamonds marking the local midday on each track.


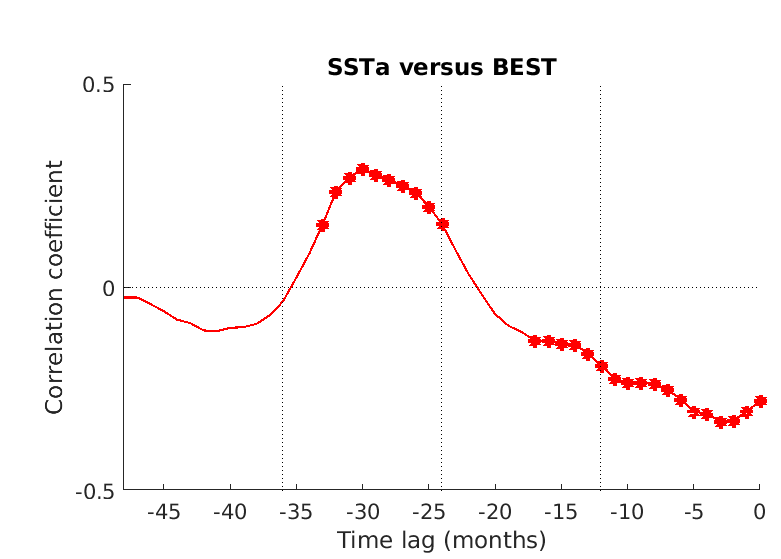


**Figure S2**. Correlations between the BEST (Bivariate El Niño Southern Oscillation) index and mean southwest Atlantic Sea surface temperature anomaly (SSTa) for time lags from 48 to 0 months (BEST preceding SSTa).


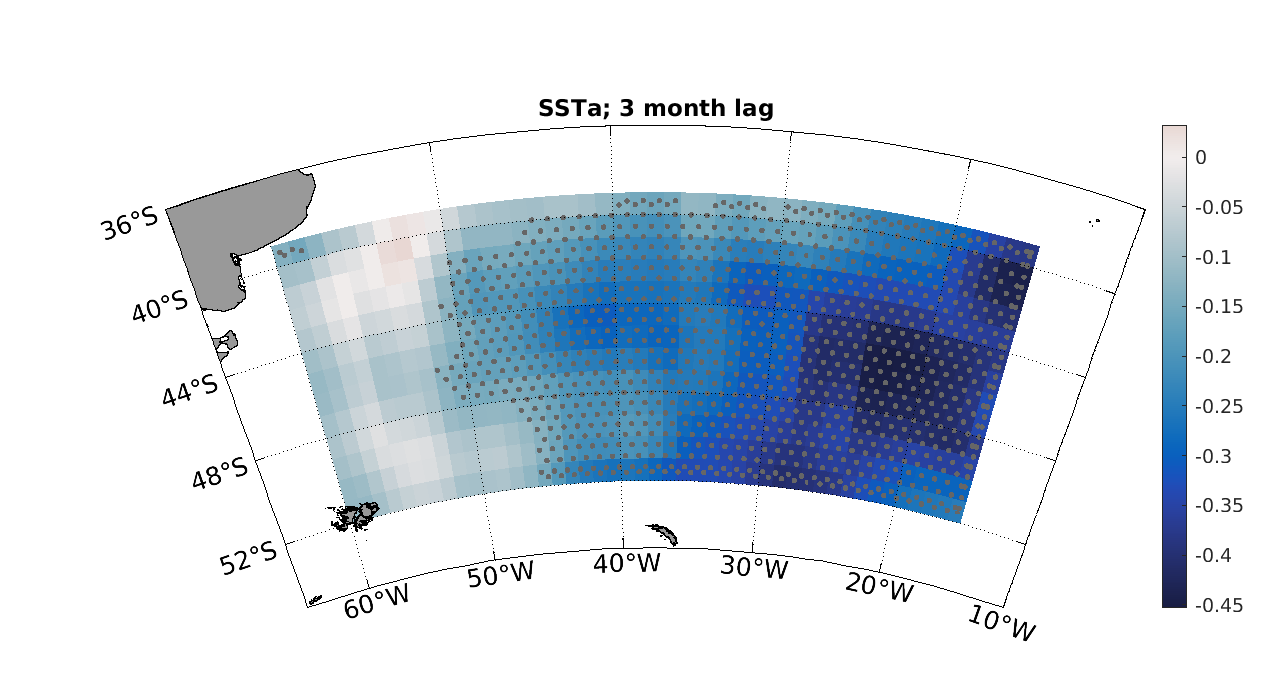


**Figure S3**. Spatial pattern of correlations between monthly mean sea surface temperature anomaly (SSTa) and the BEST Index (Bivariate El Niño Southern Oscillation) 3 months earlier. Shading is correlation (r) and grey dots indicate significant negative correlations (p < 0.005).


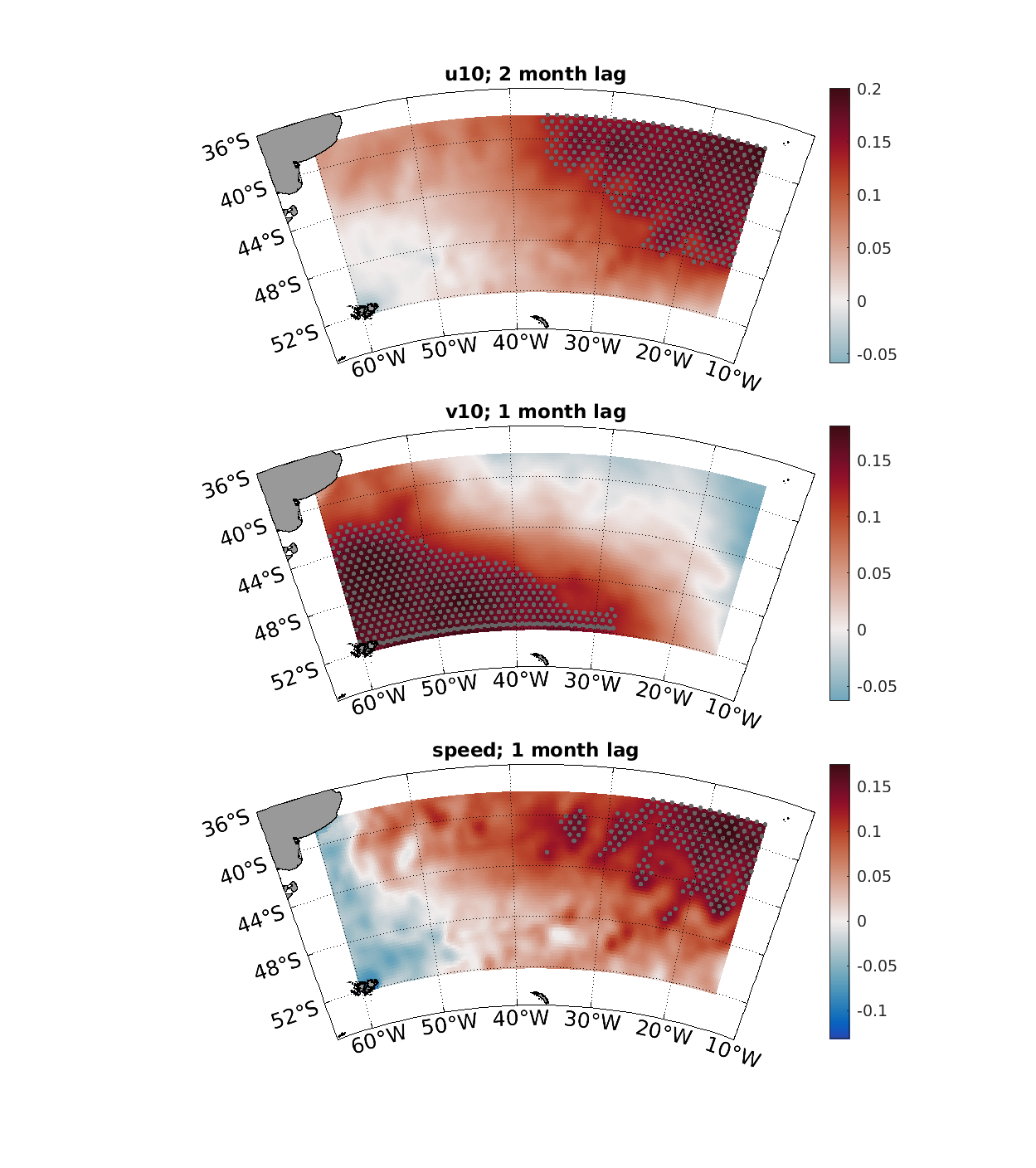


**Figure S4**. Spatial pattern of correlations between monthly mean winds (at 10 m altitude) from the ERA5 reanalysis (u10, westerly wind component; v10, southerly wind component; speed, wind speed) and the BEST Index (Bivariate El Niño Southern Oscillation) for time lags of 2 months (u10) or 1 month (v10, speed), BEST preceding wind variable. Shading is correlation (r) and grey dots indicate significant correlations (p < 0.005).

**
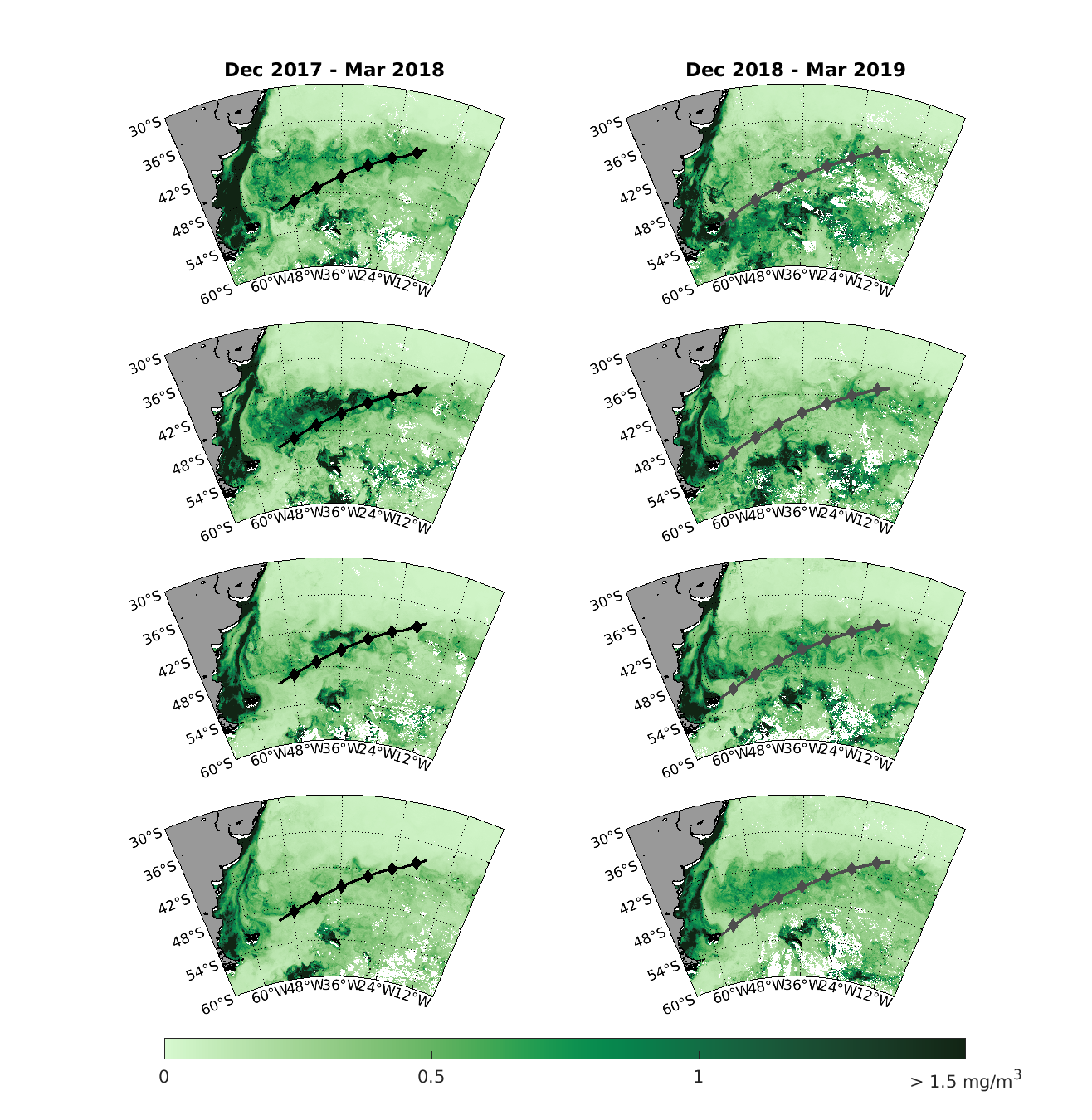
**

**Figure S5.** Monthly progression of mean surface Chlorohyll *a* prior to and contemporaneous with the cruises in 2018 and 2019 (see methods for details). Cruise tracks, when fisheries acoustics were active, are shown as black (2018) and grey (2019) lines, with diamonds marking the local midday on each track.


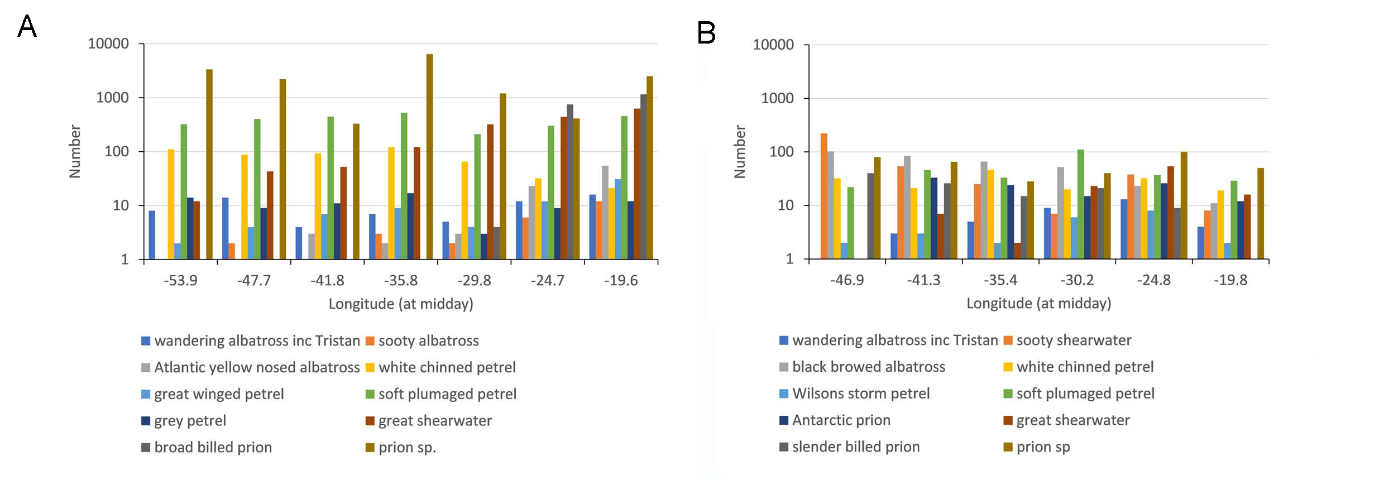


Fig. S6. Number of the 10 most abundant bird species observed along the cruise tracks, A in 2018 and B in 2019. The observations are plotted against local midday longitude for each day.

**
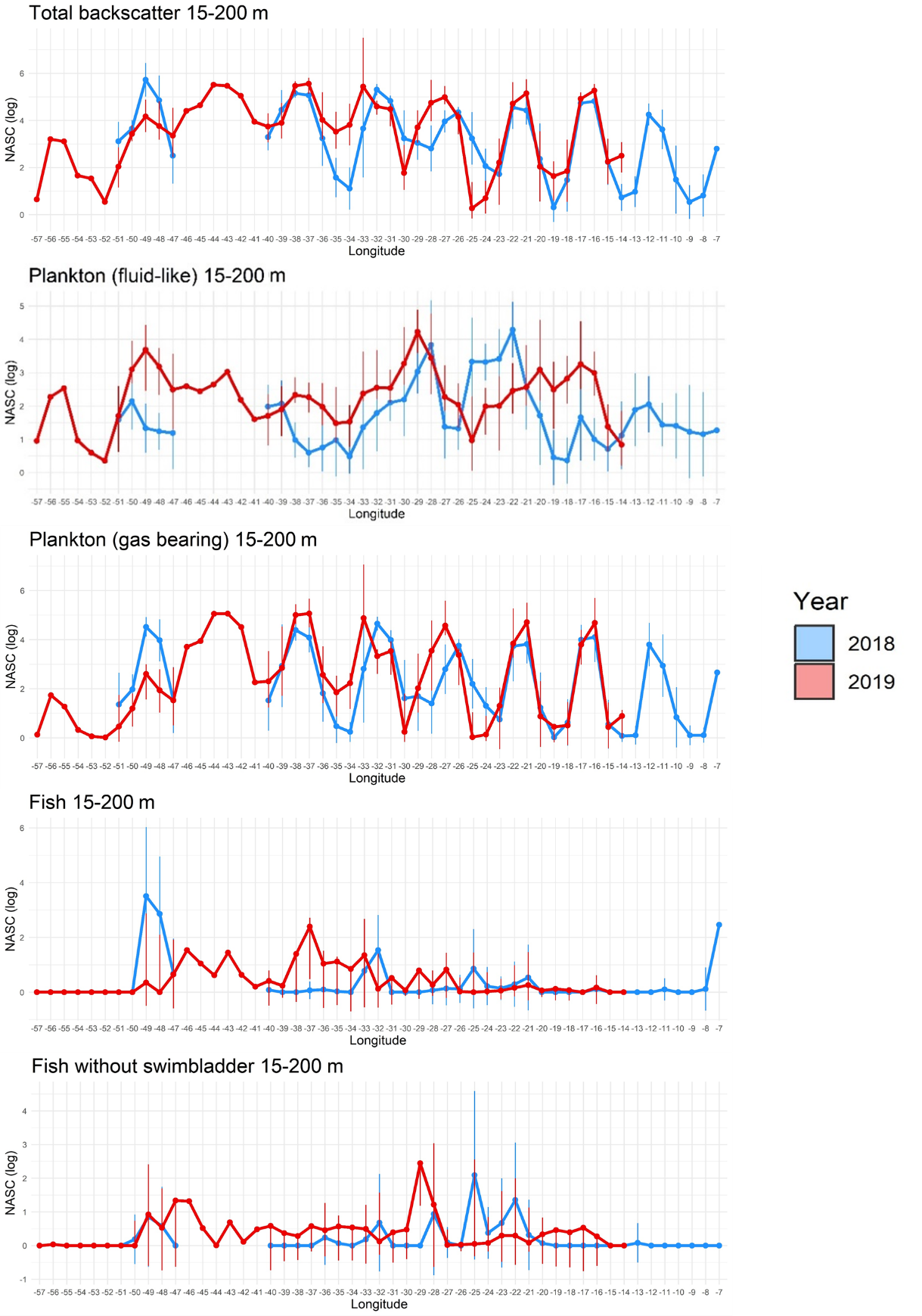
**

**Figure S7** - Acoustic backscatter of the 15 to 200 m depth layer separated by categories (total, fluid-like plankton, gas-filled plankton, fish with swimbladder, fish without swimbladder) used as proxy for biomass detected during the transit from the Falkland Islands to Tristan da Cunha on both the JCR (2018) and DY100 (2019) cruises.

**
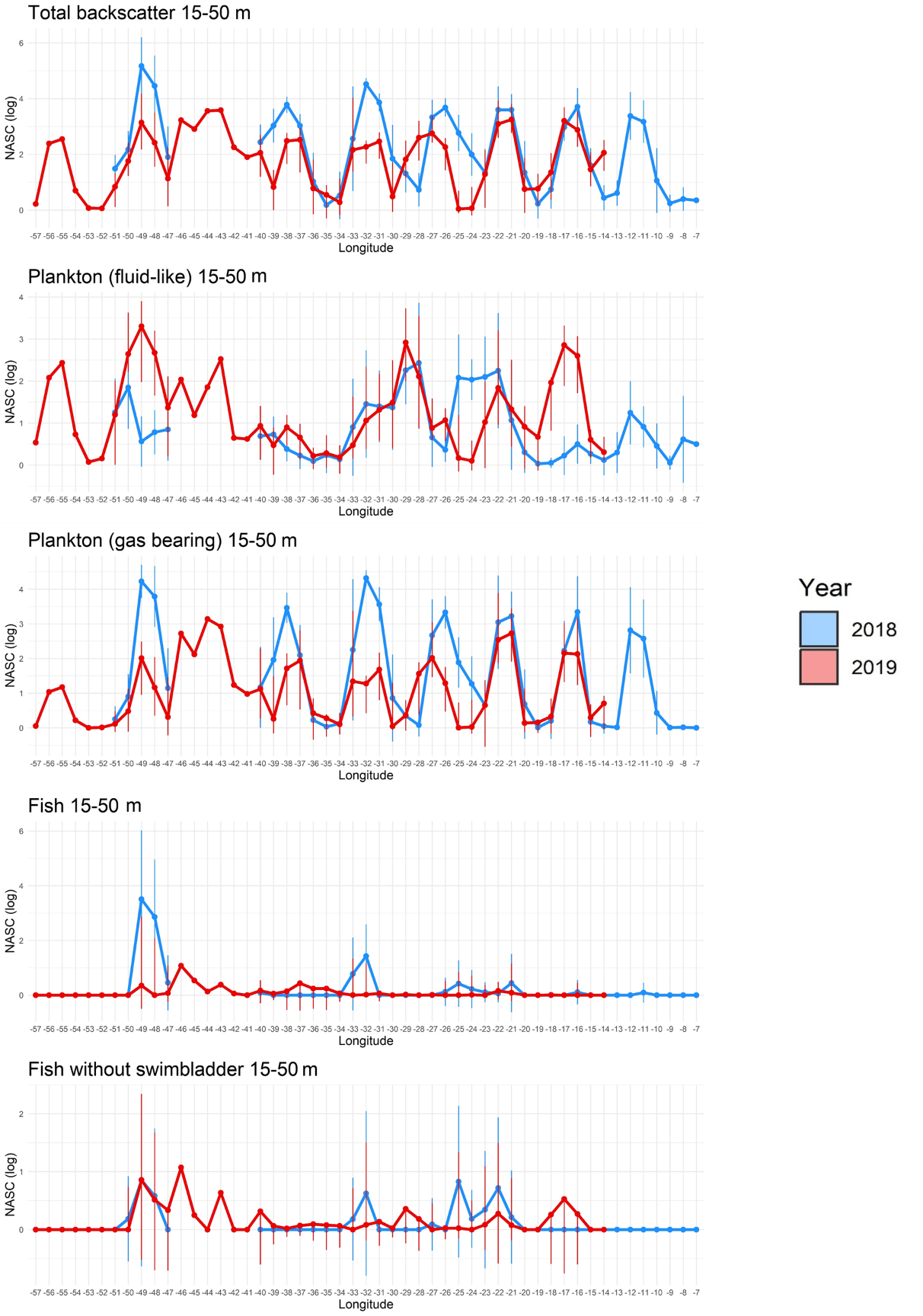
**

**Figure S8** - Acoustic backscatter of the 15 to 50 m depth layer separated by categories (total, fluid-like plankton, gas-filled plankton, fish with swimbladder, fish without swimbladder) used as proxy for biomass detected during the transit from the Falkland Islands to Tristan da Cunha on both the JCR (2018) and DY100 (2019) cruises.


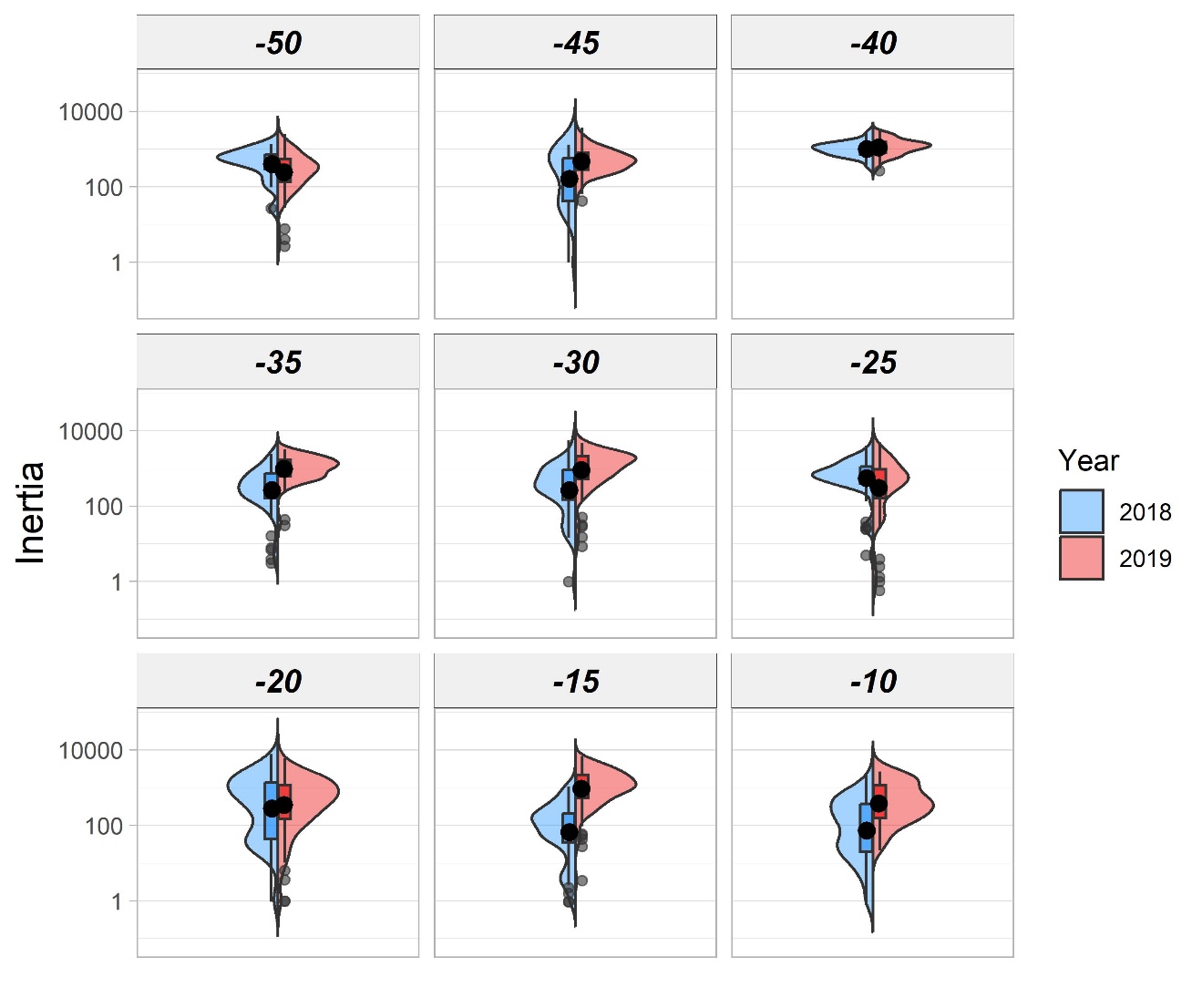


**Figure S9** - Violin plots of inertia of total backscatter at 70 kHz recorded during daytime across longitude.

**Table S1.** Combinations of daily mean sea surface temperature (SST), Chlorophyll a (Chl a) and fisheries acoustics (NASC 0-50m) yielding the best matches to the bird assemblage data in each year, as measured by a Spearman’s rank correlation (Ps). Calculated using BEST Biota-environment (BIOENV) procedure (Primer v.7).

| Ps | Correlate | P |
| --- | --- | --- |
| 0.551 | SST |  |
| 0.460 | SST, NASC50 |  |
| 0.399 | SST, Chl a, NASC50 |  |
|  |  | 0.014 |

**Table S2**. Acoustic parameters and calibration settings used for the survey conducted on the *RRS James Clark Ross* in 2018.

| **Variable** | **18 kHz** | **38 kHz** | **70 kHz** | **120 kHz** | **200 kHz** | **333 kHz** |
| --- | --- | --- | --- | --- | --- | --- |
| Transducer type | ES18-11 | ES38B | ES70-7C | ES120-7C | ES200-7C | ES333-7C |
| Ping rate (pings/s) | 2-4 | 2-4 | 2-4 | 2-4 | 2-4 | 2-4 |
| Transducer power (W) | 1400 | 1000 | 750 | 250 | 150 | 50 |
| Pulse length (us) | 1024 | 1024 | 1024 | 1024 | 1024 | 1024 |
| 2-way beam angle (dB) | -17.1 | -20.7 | -20.5 | -20.4 | -20.3 | -20.3 |
| Transducer gain (dB) | 23.10 | 23.39 | 27.03 | 26.94 | 27.03 | 25.10 |
| Sa correction (dB) | -0.67 | -0.046 | 0.013 | 0.062 | -0.39 | -0.64 |
| 3dB beam along (°) | 10.93 | 6.92 | 6.82 | 6.55 | 6.53 | 6.71 |
| 3dB beam athwart (°) | 10.89 | 6.95 | 6.73 | 6.59 | 6.31 | 6.76 |

**Table S3.** Acoustic parameters and calibration settings used for the survey conducted on the *RSS Discovery* in 2019.

| **Variable** | **38 kHz** | **70 kHz** | **120 kHz** | **200 kHz** |
| --- | --- | --- | --- | --- |
| Transducer type | ES38B | ES70-7C | ES120-7C | ES200-7C |
| Ping rate (pings/s) | 2-4 | 2-4 | 2-4 | 2-4 |
| Transducer power (W) | 2000 | 750 | 250 | 300 |
| Pulse length (us) | 1024 | 1024 | 1024 | 1024 |
| 2-way beam angle (dB) | -20.7 | -20.5 | -20.4 | -19.6 |
| Transducer gain (dB) | 25.66 | 26.47 | 23.4 | 21.87 |
| Sa correction (dB) | -0.52 | -0.37 | -0.24 | -0.30 |
| 3dB beam along (°) | 7.03 | 6.52 | 6.26 | 6.54 |
| 3dB beam athwart (°) | 6.98 | 6.54 | 6.27 | 6.56 |

**Table S4.** dB-differencing values used for species/groups acoustic discrimination.

| **Categories** | **Classes** | **ΔMVBS120–70** | **Example** |
| --- | --- | --- | --- |
| Fish | Fish with swimbladder | < 2 |  |
|  | Fish without swimbladder | > 2 |  |
| Plankton | Fluid-like plankton | > 2 | euphausiids, copepods |
|  | Gas-bearing plankton | < 2 | gelatinous and gas-bearing siphonophores, fish larvae |

**Table S5**. Whale and Bird observation effort on the 2018 (JCR) and 2019 (DY100) cruises

| Cruise | Date | Start Lat | Start Long | End Lat | End Long | Distance nmi |
| --- | --- | --- | --- | --- | --- | --- |
| JCR | 15/03/2018 | -50.78 | -54.14 | -50.09 | -51.79 | 99 |
| JCR | 16/03/2018 | -49.02 | -48.21 | -48.66 | -46.77 | 61 |
| JCR | 17/03/2018 | -47.19 | -42.20 | -46.61 | -40.37 | 82 |
| JCR | 18/03/2018 | -45.16 | -35.84 | -44.58 | -34.06 | 83 |
| JCR | 19/03/2018 | -43.36 | -30.36 | -42.74 | -28.52 | 89 |
| JCR | 20/03/2018 | -41.62 | -25.22 |  |  | 0 |
| JCR | 21/03/2018 | -40.02 | -20.13 | -39.36 | -18.52 | 84 |
| DY100 | 14/03/2019 | -48.84 | 47.91 | 48.15 | -45.61 | 100 |
| DY100 | 15/03/2019 | -47.13 | 42.25 | 42.48 | -40.21 | 92 |
| DY100 | 16/03/2019 | -45.3 | 36.46 | 44.75 | -34.78 | 78 |
| DY100 | 17/03/2019 | -43.57 | 31.13 | 42.88 | -29.06 | 100 |
| DY100 | 18/03/2019 | -41.74 | 25.66 | 41.2 | -24.1 | 77 |
| DY100 | 19/03/2019 | -40.01 | 20.64 | 39.51 | -18.99 | 82 |
